# Preparation of Selenium-Based Drug-Modified Polymeric Ligand-Functionalised Fe_3_O_4_ Nanoparticles as Multimodal Drug Carrier and Magnetic Hyperthermia Inductor

**DOI:** 10.3390/ph16070949

**Published:** 2023-06-30

**Authors:** Itziar Galarreta-Rodriguez, Mikel Etxebeste-Mitxeltorena, Esther Moreno, Daniel Plano, Carmen Sanmartín, Saad Megahed, Neus Feliu, Wolfgang J. Parak, Eneko Garaio, Izaskun Gil de Muro, Luis Lezama, Idoia Ruiz de Larramendi, Maite Insausti

**Affiliations:** 1 Departamento Química Orgánica e Inorgánica, Facultad de Ciencia y Tecnología, University of the Basque Country (UPV/EHU), Sarriena s/n, 48940 Leioa, Spain; itziar.galarreta@esrf.fr (I.G.-R.); izaskun.gildemuro@ehu.eus (I.G.d.M.); luis.lezama@ehu.es (L.L.); idoia.ruizdelarramendi@ehu.eus (I.R.d.L.); 2 Department of Pharmaceutical Technology and Chemistry, University of Navarra, Irunlarrea 1, 31008 Pamplona, Spain; mikel.etxebeste@cib.csic.es (M.E.-M.); dplano@unav.es (D.P.); sanmartin@unav.es (C.S.); 3 The Navarra Medical Research Institute (IdiSNA), Irunlarrea 3, 31008 Pamplona, Spain; 4 Tropical Health Institute of the University of Navarra (ISTUN), University of Navarra, Irunlarrea 1, 31008 Pamplona, Spain; emorenoa@unav.es; 5 Fachbereich Physik, Universität Hamburg, 22761 Hamburg, Germanywolfgang.parak@uni-hamburg.de (W.J.P.); 6 Physics Department, Faculty of Science, Al-Azhar University, Cairo 11884, Egypt; 7 Center for Applied Nanotechnology CAN, Fraunhofer Institute for Applied Polymer Research IAP, 20146 Hamburg, Germany; 8 Departamento de Ciencias, Universidad Pública de Navarra, Campus Arrosadía, 31006 Pamplona, Spain; eneko.garayo@unavarra.es; 9 Institute for Advanced Materials and Mathematics (INAMAT2), Universidad Pública de Navarra, Campus de Arrosadía, 31006 Pamplona, Spain; 10 BCMaterials, Basque Center for Materials, Applications and Nanostructures, UPV/EHU Science Park, 48940 Leioa, Spain

**Keywords:** magnetic nanoparticles, drug carriers, magnetic hyperthermia inductor

## Abstract

In recent years, much effort has been invested into developing multifunctional drug delivery systems to overcome the drawbacks of conventional carriers. Magnetic nanoparticles are not generally used as carriers but can be functionalised with several different biomolecules and their size can be tailored to present a hyperthermia response, allowing for the design of multifunctional systems which can be active in therapies. In this work, we have designed a drug carrier nanosystem based on Fe_3_O_4_ nanoparticles with large heating power and 4-amino-2-pentylselenoquinazoline as an attached drug that exhibits oxidative properties and high selectivity against a variety of cancer malignant cells. For this propose, two samples composed of homogeneous Fe_3_O_4_ nanoparticles (NPs) with different sizes, shapes, and magnetic properties have been synthesised and characterised. The surface modification of the prepared Fe_3_O_4_ nanoparticles has been developed using copolymers composed of poly(ethylene-alt-maleic anhydride), dodecylamine, polyethylene glycol and the drug 4-amino-2-pentylselenoquinazoline. The obtained nanosystems were properly characterised. Their in vitro efficacy in colon cancer cells and as magnetic hyperthermia inductors was analysed, thereby leaving the door open for their potential application as multimodal agents.

## 1. Introduction

The use of magnetic nanoparticles (NPs) in biomedical research applications has increased significantly [1]. In fact, they are currently being studied not only in traditional clinical detection therapies such as magnetic resonance imaging (MRI), but also as drug delivery systems or therapeutic agents for cancer treatment because of their capacity of heating induction upon exposure to an alternating current (AC) magnetic field [2,3,4]. This magnetic hyperthermia therapy could be an alternative for particular cancers with difficulty to treat with surgery. Such is the case of colorectal cancer, a type of disease with a high incidence in men and in women, which very frequently is diagnosed at an advanced stage when it has evolved into a metastatic stage. The most common treatment for the disease is chemotherapy [5]. Regardless of the fact that the aim of hyperthermia heating is to damage tumour cells, currently hyperthermia appears rather to be a strategy to enhance chemotherapy or radiotherapy [6,7]. The biological effects of heating, as an increase in oxidative stress, ion permeability and blood flow, the inhibition of DNA repair mechanisms, the activation of natural-killer and dendritic cells, changes in organisation of the cytoskeleton, etc., improve chemotherapy delivery and reinforce radiotherapy [8,9]. Despite the growth in the number of studies about magnetic NPs for applications in hyperthermia, clinical implementation is lacking due to the technological challenges related to the design and approval of an effective composition of a magnetic system with optimal physical characteristics and improved heating efficiency [10,11]. 

One of the most interesting compositions for magnetic hyperthermia is magnetite, Fe_3_O_4_. The biocompatibility, biodegradability, high magnetisation, suitable magnetic anisotropy and versatility of the size and shape of this compound make this material very promising as an inductor of magnetic hyperthermia [12]. In addition, iron oxide NPs can be functionalised with different drugs such as doxorubicin, bortezomib, cisplatin, irinotecan, etc., or can be directly incorporated in nanocarriers in the form of micelles, liposomes, or polymers. The potential of Fe_3_O_4_ NPs to enhance delivery and cytotoxicity might overcome the limitations of conventional carriers [13,14]. 

Drug derivatives of quinazoline have shown anti-inflammatory, antibacterial, anticonvulsant, or anticancer activity, being able to interact with different targets [15,16,17,18]. The preparation and the biological activity in different cancer lines of 2-seleno-4-amino and 2,4-diseleno-derivatives of quinazoline have been previously reported and their antiproliferative activity has been proven, being compared to etoposide and cisplatin [19]. The choice of selenium as a linker is related to the interest in it as an anti-cancer agent due to its oxidative properties and high selectivity against a variety of malignant cells, compared to those of traditional chemotherapeutics [20]. It has been reported that selenium can inhibit the proliferation of adenocarcinoma-associated colon cancer in mice [21]. However, many selenium compounds present low water solubility [22,23]. By attaching selenium compounds to hydrophilic NPs, their water solubility can be enhanced. 

However, in spite of the thorough biological activity of Se-containing compounds by themselves or in combination with other therapeutic agents, not many attempts have been made to analyse the biological effects of NPs modified with organoselenium compounds. In fact, the scarce literature points out the improvement obtained for compositions of gold NPs functionalised with selenium derivatives. A cell growth inhibition of 50% with a lower content of Se was observed for functionalised compositions compared to that of free drugs [24].

So, the aim of this work was to create an effective nanosystem for drug delivery and hyperthermia response for application in treatments of colorectal cancer. The 4-amino-2-pentylselenoquinazoline (EM102) drug has been incorporated in two specific copolymer ligands for the functionalisation of Fe_3_O_4_ NPs. 

Iron(II,III) oxide NPs have been obtained from the thermal decomposition of an iron(III) oleate precursor. Since these Fe_3_O_4_ NPs are coated by oleic acid, they are hydrophobic. For making them hydrosoluble and for properly anchoring biomolecule drugs, they have been functionalised with an amphiphilic copolymer, poly(ethylene-alt-maleic anhydride) (PMA) conjugated with dodecylamine (DDA) [25,26,27]. This PMA copolymer is formed from alternating up and down maleic anhydride rings linked by butylene groups. The anhydrous rings in the monomer can spontaneously react with amino-compounds and yield an amide bond and a free carboxylic acid [28]. In this way, we have used PMA to conjugate with the 4-amino-2-pentylselenoquinazoline (EM102) drug and to design the drug carrier nanosystem. Furthermore, with the aim of lengthening the lifetime in the bloodstream and improving the dispersion of the system in biological media, polyethylene glycol (PEG) molecules have also been incorporated on the surface of the nanoparticle [29,30].

## 2. Results and Discussion

### 2.1. Influence of Synthesis Parameters on the Structural, Morphological and Magnetic Properties of Fe_3_O_4_ Nanoparticles

The thermal decomposition of iron(III) oleate in a mixture of 1-octadecece, dibenzyl ether, and oleic acid yielded iron oxide NPs of 13 nm (Fe_3_O_4__A sample) and 18 nm (Fe_3_O_4__B sample) (Table 1). A mixture of ODE and DBE organic solvents was used to avoid the appearance of the metastable wüstite phase in the prepared NPs and to induce different growing behaviours [31].

**Figure 1:**
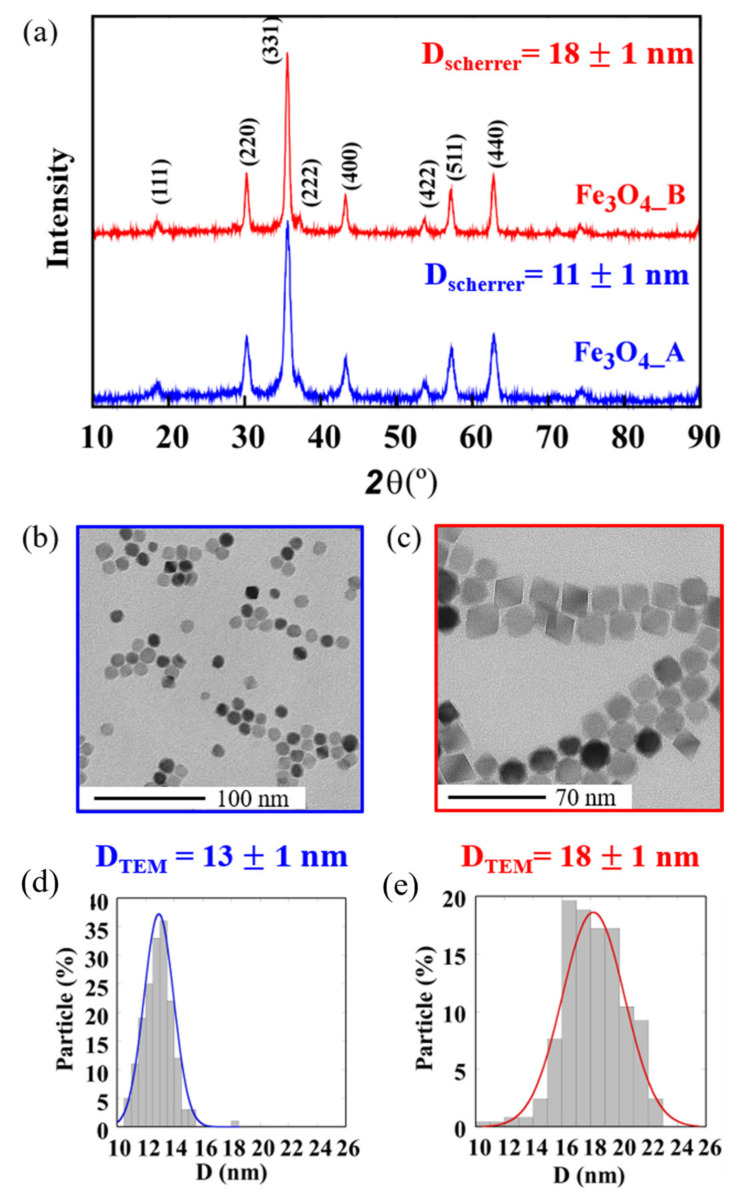
(**a**) XRD patterns, (**b**,**c**) TEM micrographs and (**d**,**e**) size distributions of the diameters of sample (**a**,**b**,**d**) Fe_3_O_4__A and sample (**a**,**c**,**e**) Fe_3_O_4__B.

The X-ray diffractograms measured at room temperature are shown in Figure 1a. The positions and intensities of diffraction maxima match well with the structure of magnetite (inverse spinel *F*d-3m, JPCDS n° 89-0691) with no trace of secondary phases. The crystallite sizes of the prepared Fe_3_O_4__A and Fe_3_O_4__B samples were obtained from the (311) main diffraction peak by means of Scherrer’s equation (E1 in the electronic Supporting Information, ESI, and Table 1) [32] and compared with those observed via TEM analysis. The Fe_3_O_4__A sample presents larger peak widths than the Fe_3_O_4__B sample does, indicating a smaller size (11 ± 1 nm) than that of the Fe_3_O_4__B NPs (18 ± 1 nm) [33]. The presence of a larger volume of ODE would induce higher reflux temperatures (see Table 1), which could derive in a higher iron species diffusion in the reaction flask, favouring nanoparticle growth [34]. 

Figure 1b–f shows TEM images of the Fe_3_O_4__A and Fe_3_O_4__B samples with the corresponding size distributions. The measured core diameters are in agreement with those previously calculated from the XRD data (Table 1), meaning that the NPs are mainly composed of single crystals. The d-spacings obtained from electron diffraction patterns are also in good accord with those from the magnetite structure (Figure S2 in ESI). Fe_3_O_4__A NPs (Figure 1b), with a cuboctahedral morphology, present the smallest sizes, 13 ± 1 nm while the Fe_3_O_4__B sample is composed of 18 ± 2 nm NPs with an octahedral morphology. The surface energy of the NPs determines the location of the ligands on specific faces, preventing their growth [35]. In spinel-type structures, the {100} faces present lower surface energy than do the {111} ones [36]. Thus, for higher iron precursor concentrations, growing process will be favoured in some planes and for a low amount of precursors, more regular growth at a lower speed will occur, creating more spherical NPs [37]. In addition, a mixture of solvents with lower reflux temperatures results in the slower growth of NPs as at higher temperatures Fe(oleate)_3_ decomposition is favoured [34]. These decomposition processes yield NPs surrounded by oleic acid which guarantees the stability of the NPs in dispersion. The amount of ligand coating has been calculated via thermogravimetry analysis (Table 1. Figure S3 in ESI).

Nevertheless, at this point we must remember that in X-ray diffraction both Fe_3_O_4_ and γ-Fe_2_O_3_ present very similar diffraction patterns and other techniques such as as Mössbauer, XPS or VSM measurements are neccessary to discard maghemite in the samples. This fact is going to be subsequently discussed in the next section. Nevertheless, it is noteworthy that thermal decomposition methods from metallo-organic precursors favour the presence of magnetite, being the most reliable one with which to obtain Fe_3_O_4_ nanoparticles below 10 nm [38], and for bigger particles oleate precursors with proper control of the mixture of solvents yield stoichiometric phases [12].

### 2.2. Magnetic Properties of the Prepared Magnetite Nanoparticles

**Figure 2:**
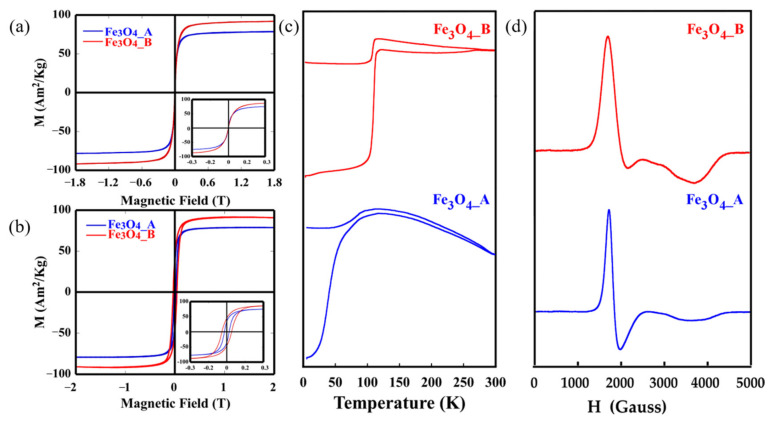
Hysteresis cycles at RT (**a**), hysteresis cycles at 5K normalised to RT M_s_ values (**b**), magnetisation as a function of temperature after cooling at zero field and at 10 Oe field (**c**) and electron magnetic resonance spectroscopy measurements at RT of Fe_3_O_4__A and Fe_3_O_4__B (**d**).

The effects of the size and shape of the magnetite NPs on their magnetic behaviour were analysed by means of magnetisation measurements versus magnetic field, M(H), and temperature, M(T), as variables. In Figure 2a, hysteresis loops at room temperature are presented, where the magnetisation reaches values close to saturation at moderately low fields (<5 kOe), in good accord with the ferrimagnetic character of magnetite. The saturation magnetisation (M_s_) of the samples Fe_3_O_4__A and Fe_3_O_4__B at 300 K are 78 Am^2^ kg^−1^ and 92 Am^2^ kg^−1^ (referring to the mass of Fe_3_O_4_), respectively (Table 2). These values have been normalised per unit mass of the Fe_3_O_4_ cores, which were calculated via thermogravimetry by subtracting the organic content from the total particles mass. The saturation value of sample Fe_3_O_4__B, close to the magnetic saturation of bulk magnetite, is indicative of the high purity and crystallinity of the Fe_3_O_4_ phase obtained by controlling the thermal decomposition process, as was already deduced from the TEM measurements [39]. The lower values of saturation in sample Fe_3_O_4__A can be ascribed to surface-volume effects, which are more pronounced in smaller-sized NPs. The large proportion of atoms on the nanoparticle surface could cause reduced ferrimagnetic coordination, inducing surface magnetic frustration and reduced electron mobility [40,41]. 

The dipolar and magnetic interactions usually present between the nanoparticles disrupt magnetisation at low applied magnetic fields. In order to minimize the interactions at 5 K, M vs. H measurements were performed on colloidal dispersions of NPs in chloroform (0.05 mg·mL^−1^; mass referring to Fe_3_O_4_) deposited on a filter paper. The coercive field values at 5 K were obtained by normalising these hysteresis cycles to the magnetisation obtained at room temperature (Figure 2b, Table 2). The value of 40 mT for sample Fe_3_O_4__B is typical of ferrimagnetic NPs of Fe_3_O_4_ with diameters near 20 nm [42]. In this case, the reduced remanence value (M_r_/M_s_) is close to 0.5, which is the value predicted using the model of Stoner–Wohlfarth for uniaxial single domains with no magnetic interactions between the particles [43]. 

Magnetisation measurements have been carried out by the varying temperature after cooling in a zero field (ZFC curve) and 100 Oe magnetic field (FC curve), as shown in Figure 2c. Two different behaviours can be distinguished. The Fe_3_O_4__A sample presents a broad maximum in the ZFC curve with progressive decay at higher temperatures and the irreversibility of the FC curve, which highlights the thermal hysteresis of the NPs above this temperature (T_B_, blocking temperature) due to their superparamagnetic character [44,45]. In addition, the FC curve of Fe_3_O_4__A NPs shows a small peak around 110 K related to a soft Verwey transition which denotes the hallmark of magnetite [46]. This transition is better observed in the Fe_3_O_4__B sample. The change in the crystalline structure of magnetite and, consequently, in electron mobility induces this pronounced phase transition around 120 K (T_v_), [47]. The presence of this transition in magnetite NPs is related to the morphology. Octahedral NPs with more energetically stable (111) planes present a lower tendency to undergo surface oxidation, fewer surface effects and thus, better stoichiometry. So, the transition temperature can be used to visualize deviations from the stoichiometry in magnetites, as it occurs in the Fe_3_O_4__B sample with a T_v_ temperature of 112 K which slightly differs from that of 120 K [12]. This deviation is more pronounced in the Fe_3_O_4__A phase, where more deficiencies in stoichiometry would appear, resulting in the decrease in magnetic saturation. The interactions of oleic acid ligands on the surface layer of these smaller NPs would also affect these deviations in stoichiometry, and, consequently the loss of saturation [48].

The EMR technique has also proven to be a good complement to deepen the magnetic character of the samples and to evaluate the homogeneity and size distribution of the magnetic NPs, due to the variations in the shape, width, and resonant field of the observed signals. Figure 2d shows the spectra obtained at room temperature for the prepared Fe_3_O_4__A and Fe_3_O_4__B samples. The EMR signals are asymmetric and complex with lines of minor intensity that present different positions and widths. The values of $g_{eff}$ have been calculated from the maximum of the microwave absorption curve which corresponds with the resonant field (Table 2). The peak-to-peak line widths also appear in the table and have been determined from the positions of the maximum and minimum main signal strengths.

The calculated $g_{eff}$ values of the Fe_3_O_4__A and Fe_3_O_4__B samples move from the $g_{eff}\;$= 2 value which corresponds to that of magnetite in the paramagnetic state, being higher than that of single-crystalline magnetite at 300 K in the direction of the axis of easy magnetisation ($g_{eff\;}$= 2.122) [49]. This fact corroborates that the Fe_3_O_4__A and Fe_3_O_4__B samples are not purely superparamagnetic at room temperature [50]. The Fe_3_O_4__A and Fe_3_O_4__B samples show similar characteristics, with very low resonant fields (1840 and 2040 Gauss approx.) and reduced linewidths (260 and 470 Gauss approx.). The data are in good accord with the homogeneous distribution of sizes and shapes, and strong anisotropy in the NPs (as observed in TEM), together with the ability of the magnetic moments to easily align with the applied magnetic field [51]. Around 3700 Gauss, a secondary component appears in both spectra, more clearly identified in sample Fe_3_O_4__B, which can be ascribed to a progressive agglomeration of the particles in solution. It can be concluded that the different signals observed are mainly related to the ability of NPs to order themselves towards the applied field. 

### 2.3. Characterisation of Fe_3_O_4_ Nanoparticles Functionalised with Selenium-Based Drugs

**Figure 3:**
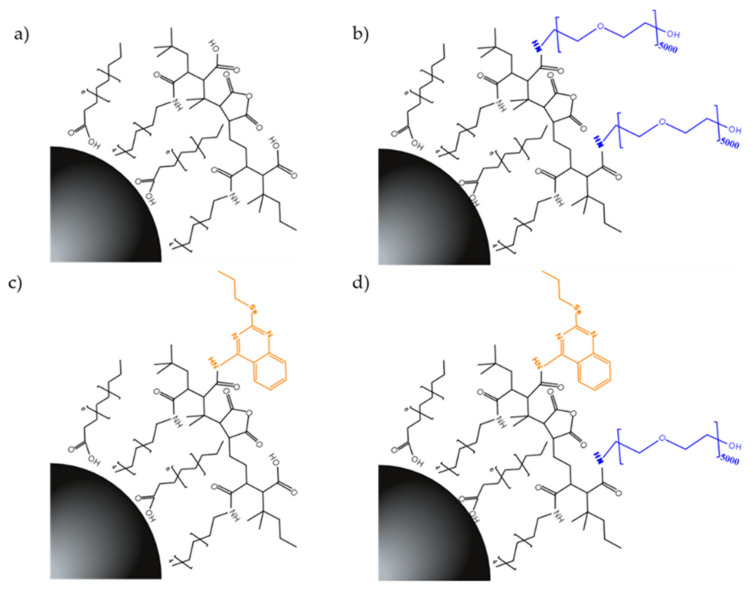
Nanosystems developed via the functionalisation of Fe_3_O_4__A and Fe_3_O_4__B NPs with (**a**) PD-, (**b**) PD-PEG-, (**c**) PD-EM102-, and (**d**) PD-EM102-PEG-prepared ligands.

In order to analyse and to establish correlations among drug carries NPs, different systems were prepared. Fe_3_O_4__A and Fe_3_O_4__B hydrophophic NPs were firstly coated by a copolymer formed using poly(isobutylene-alt-maleic anhydride) and dodecylamine, obtaining Fe_3_O_4_@PD. To improve the biocompatibility of the systems, PEG was also included in the polymer and Fe_3_O_4_@PD-PEG systems were synthesised. Finally, functionalisation with 4-amino-2-pentylselenoquinazoline (EM102 drug) was performed and Fe_3_O_4_@PD-EM102-PEG phases were obtained. In order to analyse the possible influence of PEG in the effect of the drug, the Fe_3_O_4_@PD-EM102 nanosystem without PEG was also prepared. The different nanosystems in the functionalisation process are represented in Figure 3. 

The structural differences of the copolymer-functionalised nanosystems were analysed via FTIR. In Figures S4–S8 of the ESI the synthesised PD, PD-PEG, PD-EM102, and PD-EM102-PEG copolymers are compared to Fe_3_O_4__A and Fe_3_O_4__B NPs functionalised with the copolymers, which corroborate the progressive addition of ligands to the NPs. Thus, the 3283 cm^−1^ band corresponding to the N-H stretching of secondary amines is characteristic of PD copolymer-functionalised NPs (Figure S5). The *ν*_C=O_ stretching band at 1795 cm^−1^ of the maleic anhydrous rings observed in the PD copolymer disappears in samples Fe_3_O_4_@PD and Fe_3_O_4_@PD-EM102, because of the hydrolysis of the sample and the ring opening creating carboxylic acids or -CONH- bonds with dodecylamine molecules. The broad absorption band in the 3500–3000 cm^−1^ region is due to the presence of polyethylene glycol in the PD-PEG functionalised NPs and corresponds to the stretching vibrations of hydroxyl groups and to the asymmetric stretching vibration of *ν*_CO_ and *ν*_C-O-C_ bands of the ester groups of the PEG molecule around 1260 cm^−1^ [52,53,54,55]. 

Nevertheless, for the drug-functionalised particles (Fe_3_O_4_@PD-EM102 and Fe_3_O_4_@PD-EM102-PEG12.5%), it has not been possible to detect the *ν*_C=C_ and *ν*_C=N_ stretching bands at 1645 cm^−1^ and the aromatic *ν*_C-H_ stretching band at 3141 cm^−1^ characteristic of the EM102 drug [53]. The low proportion of EM102 (5% of mmol percent of PMA) linked to the PD copolymer chains hampers the detection of this drug in the samples. As a consequence, the presence of selenium in drug-functionalised samples was analysed via ICP-AES (Table 3). It can be observed that samples containing the Fe_3_O_4__A NPs present more drug molecules (123 to 349 drug molecules/NP) than those with Fe_3_O_4__B configurations.

The change in hydrodynamic diameter due to the polymeric layer addition (PD, PD-PEG, PD-EM102 and PD-EM102-PEG) to the NPs, together with the stability of the samples (zeta potential) in distilled water (pH 7) and in phosphate-buffered saline water solution (PBS 1:10 water volume) buffer was analysed via dynamic light scattering (DLS) (Figure S9 and Table 3). Indeed, the level of aggregation of the nanosystems and their stability are key factors for implementation in biological environments [56,57]. 

According to the literature, the coating carried out with the PD polymer should produce an increase of 10 nm in the initial particle diameter [58]. Nevertheless, diameters recorded both in water and in PBS are larger than the corresponding diameter calculated via the addition of a 10 nm coating to the diameter of the Fe_3_O_4_ cores (Table 1), which suggests a certain degree of agglomeration in the functionalisation process. It is noteworthy that the nanosystems based on the smaller inorganic cores, Fe_3_O_4__A, present smaller hydrodynamic diameters than the systems formed by Fe_3_O_4__B NPs for the same recovering process and coating. Finally, the incorporation of small amounts of the EM102 molecule to the PD ligand does not significantly influence the coating process of the NPs.

Concerning the zeta potentials of the samples, it must be pointed out that potentials between −50 and −43 mV have been recorded in all analysed samples, due to the negative charges from the non-bonded acidic groups of the PMA. These values are similar to those observed in the literature for related carboxylated coatings [59,60,61]. 

### 2.4. Magnetic Hyperthermia Response

**Figure 4:**
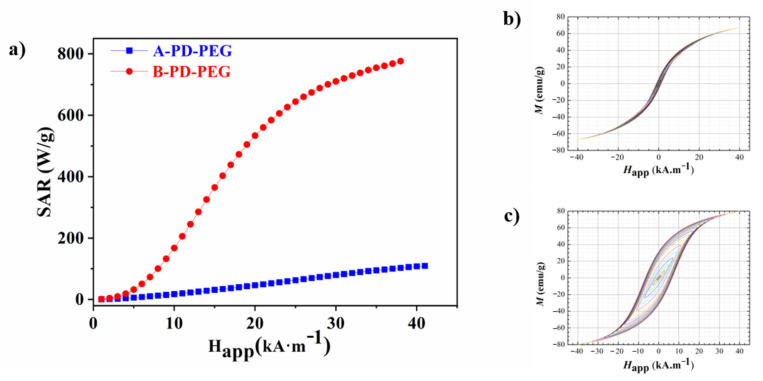
Experimental SAR versus applied field for Fe_3_O_4__A@PD-PEG (blue) and Fe_3_O_4__B@PD-PEG (red) (**a**) and AC hysteresis loops measured at 311 kH of the Fe_3_O_4__A@PD-PEG (**b**) and Fe_3_O_4__B@PD-PEG (**c**) samples.

In order to analyse the heating capacity of the NPs coated with the PD-PEG ligand, the dynamic magnetisation of the samples was obtained by via AC magnetometry. For the magnetic hyperthermia response and hysteresis loops measurement, samples dispersed in water with a mass concentration of 1 mg·mL^−1^ were measured. From these measurements, the specific absorption rate (SAR) can be calculated by computing the area (A) of the hysteresis loops according to next equation:

(1) $$SAR=-\mu_{0}\int M_{t}dH_{AC}$$

where $M_{t}$ is the time-dependent dynamic magnetisation (normalised by NP mass) and $H_{AC}$ is the externally applied magnetic field intensity. The experimental SAR values versus the applied magnetic field of the Fe_3_O_4__A@PD-PEG and Fe_3_O_4__B@PD-PEG samples are represented in Figure 4. The AC magnetic field frequency applied was 311 kHz whereas the intensity of the magnetic field was increased to 40 kA/m. 

The power absorption of the systems increases non-linearly when increasing the magnetic field amplitude, confirming significant differences between both samples but, at the same time, it demonstrates the hyperthermic capacity of both configurations. On the one hand, the Fe_3_O_4__A@PD-PEG sample reached a SAR value of 109 W·g^−1^ (the mass refers to the NP core) at the highest applied field of 40 kA/m while Fe_3_O_4__B@PD-PEG reaches a value of 776 W·g^−1^ (the mass refers to the NP core). As has been observed in the literature, the variation in the characteristics of magnetite NPs, size, shape and stoichiometry drastically change their response to the applied magnetic field [62,63]. In addition, the configuration of the NPs after polymer coating can also condition the heating performance of the system [64]. All these factors together with the variation of the measurement conditions (frequency and field amplitude) make difficult the comparison of SAR values with those observed in the literature for related iron nanoparticles [65]. As previously explained, the magnetisation of iron oxide NPs decreases with the decreasing particle size due to surface and internal spin disorders, which explains the low SAR value obtained for the Fe_3_O_4__A@PD-PEG sample [48,66]. Nevertheless, the higher SAR values in sample Fe_3_O_4__B@PD-PEG is due to the much larger effective relaxation time (τ) for this sample. The larger Néel relaxation time in Fe_3_O_4__B@PD-PEG is caused by its larger grain size compared to that of Fe_3_O_4__A@PD-PEG (18 nm and 11 nm, respectively (Table 1), as well as by its higher effective anisotropy due to its cubic shape compared to the nearly spherical shape for sample Fe_3_O_4__A@PD-PEG [62]. 

The increasing hydrodynamic diameter of the Fe_3_O_4__B@PD-PEG NPs, reflecting the agglomeration of the NPs due to the functionalisation process by means of the PD-PEG copolymer, can also explain the higher SAR value for the Fe_3_O_4__B@PD-PEG sample [67,68]. Dipolar interactions between agglomerated particles can enhance heating power when creating structures with high anisotropy, such as chains, aggregates, etc. [68,69,70]. 

### 2.5. Toxicity of Fe_3_O_4_ Nanoparticles Functionalised with Selenium-Based Drugs

**Figure 5:**
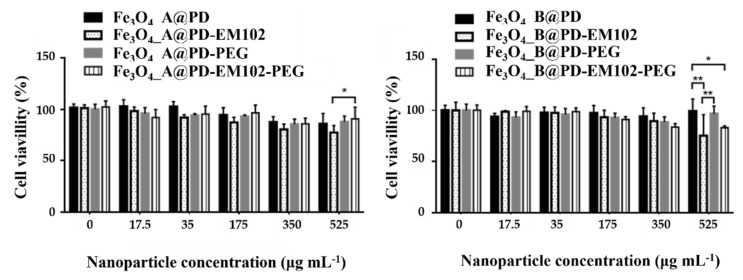
Cytotoxicity experiments for the Fe_3_O_4__A and Fe_3_O_4__B samples functionalised with PD, PD-EM102, PD-PEG%, and PD-EM102-PEG% copolymers at different concentrations of the nanosystems in HTC 116 cells. * and ** pair of samples with significant improvement in toxicity.

The efficacy of the prepared nanosystems as drug carriers in colon cancer cells, specifically in the HCT116 cell line (ATCC, American Type Culture Collection), was analysed by means of a colourimetric MTT assay to assess the metabolic activity of cells. The viabilities of drug-functionalised NPs compared to those of NPs without drugs at different concentrations are represented in Figure 5. In fact, both, the Fe_3_O_4__A and Fe_3_O_4__B NPs do not show significant cytotoxicity up to concentrations of 175 µg·mL^−1^ (the mass refers to the Fe_3_O_4_ cores) where the drug concentration in sample Fe_3_O_4__A@PD-EM102 is 645 nM and 700 nM for sample Fe_3_O_4__B@PD-EM102-PEG.

In this sense, the most concentrated samples (525 μg·mL^−1^) have showed the highest toxicity. For the samples Fe_3_O_4__A@PD-EM102 and Fe_3_O_4__B@PD-EM102, 9% and 28% lower cell viability was observed compared to those in the experiments performed with the Fe_3_O_4__A@PD and Fe_3_O_4__B@PD samples. On the other hand, no significant improvement in toxicity was observed for the PEGylated samples, Fe_3_O_4__A@PD-EM102-PEG or Fe_3_O_4__B@PD-EM102-PEG. Despite the EM102 drug concentration of 0.77 µM for Fe_3_O_4__A@PD-EM102-PEG and 0.67 µM for Fe_3_O_4__B@PD-EM102-PEG, the presence of PEG on the surface of the A@PD-EM102-PEG nanosystems could mask the activity of the EM102 molecule, i.e., the drugs might be hidden under the PEG coils, as also known for other molecules [29,71]. The presence of the PEG in both cases may create a protective layer that hinders the drug–cell interaction, and consequently, reduces the drugs’ effectiveness. In addition, many authors have shown that coating with PEG usually reduces the drug uptake by cells [71,72,73]. Anyway, the obtained results demonstrate that the presence of the EM102 drug in the nanosystems directly affects the HCT116 cell line viability, since the NPs functionalised only with PD and PD-PEG copolymers show lower toxicity. 

It has been proven for other cell lines that selenium-based compounds can prevent the proliferation of cancer cells [74,75,76]. Twelve different molecules based on selenium (using 10 µM concentrations) were tested on the HT29 colon cancer cell line and cell proliferation was reduced up to the 50% [77]. L. Schröterová et al. also used selenium compounds (concentrations in the 64–256 µM range) to reduce cell proliferation by 50% in colon lines (HT29) [78]. Thus, comparing the amounts of drugs used in both studies and considering the results of the present investigation, a reduction of 28% in cell viability with a 0.77 µM drug concentration is a promising result for future applications of these nanosystems, in particular considering the fact that a reduction in cell proliferation in general happens already at lower NP doses than does a reduction in cell viability [79]. Therefore, it is expected that despite the increase in drug concentration, without reaching the previously mentioned values of 10 µM or 64 µM, the results could be improved, reducing proliferation with a lower amount of drugs.

## 3. Materials and Methods

### 3.1. Materials

FeCl_3_.6H_2_O (99%) was purchased from Sigma-Aldrich, sodium oleate (97%) was purchased from TCI America, ethanol was purchased from Panreac S.A, poly(ethylene glycol)-amine (M_W_ = 5000) was purchased from Laysan Bio and phosphate-buffered saline (PBS) and Dulbecco’s modified Eagle medium (DMEM) were purchased from Gibco. Oleic acid (90%), dibenzyl ether (DBE) (98%), octadecene (ODE) (90%), hexane (99%), triethylamine, dodecylamine, and poly(isobutylene-alt-maleic anhydride (PMA) (M_W_ = 4000−6000 Da) were purchased from Sigma-Aldrich.

### 3.2. Methods

#### 3.2.1. Preparation of Iron(III) Oleate

The iron(III) oleate (Fe(oleate)_3_ precursor was synthesised via the optimisation of the protocol followed by R. Chen et al. [80] For this purpose, 40 mmol of FeCl_3_·6 H_2_O and 120 mmol of sodium oleate were added to a mixture of solvents formed by 80 mL of ethanol, 140 mL of hexane and 60 mL of distilled water. This mixture was heated up to 60 °C for one hour under a N_2_ atmosphere. After cooling to room temperature (RT), the organic phase with the FeOl complex was washed several times with deionised H_2_O. Finally, the synthesis reaction solvents were evaporated overnight at 110 °C obtaining a black-reddishbrown-coloured waxy solid.

#### 3.2.2. Synthesis of Fe_3_O_4_ Nanoparticles

To prepare magnetite NPs, the following procedure was followed. In a mixture of octadecene and dibenzyl ether, 5 mmol of Fe(oleate)_3_ and 10 mmol of oleic acid were dissolved (Table 1). The solution was heated up to 110 °C and maintained for 30 min under a N_2_(g) atmosphere to remove the water and oxygen left in the reaction system. Then, the mixture was heated in two steps until 320 °C under mechanical stirring; at 10 °C min^−1^ from R.T. to 190 °C and at 3 °C min^−1^ until 320 °C. The final temperature was kept for 1 h and then, the product was cooled to room temperature. The final product was cleaned via centrifugation (20,000 rpm) using tetrahydrofuran (THF) and ethanol, whereby the supernatants above the nanoparticle precipitate were discarded. The nanoparticle stock solution was prepared by dispersing the nanoparticle precipitate in chloroform and was stored in a fridge. By changing the ODE:DBE ratio, two different syntheses were carried out and the obtained NPs in the following are referred to as Fe_3_O_4__A and Fe_3_O_4__B samples.

#### 3.2.3. Synthesis of Drug-Functionalised Copolymer and Coating Procedure

The PD (PMA-DDA) amphiphilic copolymers were prepared via the modification of 75% of the maleic anhydride monomers of the poly(ethylene-alt-maleic anhydride) (PMA) backbone with dodecylamine (DDA), leaving the other 25% for the further addition of the organoselenium-derived EM102 drug (4-amino-2-pentylselenoquinazoline) and 5 kDa PEG molecules [28]. Thus, to prepare the PD copolymer, 6.4 mmol of the PMA polymer was vigorously mixed with 4.8 mmol of DDA in 20 mL of THF solution, in the presence of 1.93 mmol of triethylamine (Table S1). After heating (80 °C) the solution for several hours, the cloudy solution became clear. The solvent was evaporated and the resulting copolymer was redissolved in dry chloroform to obtain a 0.1 M PMA stock solution. Hereby, 0.1 M refers to the monomer concentration [28]. For the preparation of the organoselenium-derived EM102 drug-functionalised PD-EM102 copolymer, a similar procedure was carried out, but this time N, N-dimethylformamide (DMF) was used as a solvent due to the poor solubility of the EM102 drug in THF. Thus, 0.676 mmol of PMA polymer was vigorously mixed with 0.5 mmol of DDA in a DMF solution (20 mL) in the presence of 0.676 mmol of triethylamine and 0.03 mmol of the EM102 drug. Then, the DMF solvent was evaporated and the obtained PD-EM102 copolymer was redissolved in dry chloroform. Finally, the PEGylation of prepared copolymers was performed. Different PEG-containing copolymers were prepared due to the different size of synthetised NPs. Thus, for the functionalisation of 13 nm NPs, 12.5%wt PEG-modified PD and PD-EM102 copolymers were prepared, while 25%wt PEG-modified ligands were prepared for the functionalisation of 18 nm core diameter NPs. In order to prepare a 12.5%wt PEG-modified PD copolymer, 40.5 mg of 5 kDa PEG-amine was added to 2 mL of PD solution (5 mg·mL^−1^ PMA) and was vigorously stirred overnight. For the preparation of the other copolymers, the same procedure was carried out using the different reagent amounts described in Table S1 in ESI, Figure 3. 

In order to coat the nanoparticles, 3 mg of NPs was added to 4 mL of a polymeric solution (PD, PD-PEG, PD-EM102 or PD-EM102-PEG) using 50 monomers of PMA per nm^2^ of the effective NP surface [28]. The mixture was stirred for 30 min in an ultrasound bath, and the solvent was evaporated in a rotavapor at 60 °C. Finally, the hydrolysis of the remaining maleic anhydride groups in the polymeric surface was performed via the addition of a sodium borate buffer at pH = 9. In this way, eight different compositions were obtained which are referred to as Fe_3_O_4__A@PD, Fe_3_O_4__A@PD-PEG, Fe_3_O_4__A@PD-EM102, Fe_3_O_4__A@PD-EM102-PEG, Fe_3_O_4__B@PD, Fe_3_O_4__B@PD-PEG, Fe_3_O_4__B@PD-EM102, and Fe_3_O_4__B@PD-EM102-PEG.

#### 3.2.4. Physical, Structural and Magnetic Experimental Characterisation 

X-ray diffraction (XRD) patterns of the dried nanoparticles were determined using a PANalytical X’Pert PRO diffractometer equipped with a copper anode (operated at 40 kV and 40 mA), diffracted beam monochromator and PIXcel detector. Scans were collected in the 10−90° 2θ range with a step size of 0.02° and scan step speed of 1.25 s. 

Iron and selenium contents were determined via inductively coupled plasma–atomic emission spectroscopy (ICP-AES), using an ELAN9000 ICP-MS (Waltham, MA, USA) spectrophotometer. 

Fourier transform infrared spectroscopy (FTIR) spectra of prepared copolymers were collected using a FTIR8400S Shimadzu spectrometer in a 4000−400 cm^−1^ range using KBr pellets.

The amount of organic matter in the prepared hydrophobic NPs was determined via thermogravimetric (TGA) measurements, performed in a NETZSCH STA 449 C thermogravimetric analyser, by heating at 10 °C/min 10 mg of the sample in a dry Ar environment. 

Dynamic light scattering (DLS) and ζ potential measurements of the NPs coated with PD, PD-PEG, PD-EM102 or PD-EM102-PEG were analysed using a Zetasizer Nano-ZS (Malvern Instruments). The measurements were carried out at a controlled temperature of 25 °C after an equilibrium duration of 3 min for 0.05 mg_Fe3O4_mL^−1^ aqueous dispersions. For each sample, 10 runs of 10 s were performed with three repetitions for all samples.

Transmission electron micrographs (TEMs) of synthesised NPs were obtained using Phillips CM200 with an accelerating voltage of 200 kV, and a point resolution of 0.235 nm. This equipment provides morphology images and the corresponding crystal structures via selected area electron diffraction.

Quasi-static magnetisation measurements as a function of magnetic field M(H) and temperature measurements M(T) were carried out using a SQUID magnetometer (MPMS3, Quantum design). These measurements were performed by drying NP colloids (∼0.1 mg/mL) on semipermeable filter paper. The saturation magnetisation, M_s_, at RT and 5 K curves were obtained from dried nanoparticles and normalised per unit mass of inorganic matter by subtracting the weight percentage of organic matter determined via thermogravimetry. 

Electron magnetic resonance (EMR) experiments were carried out in chloroform dispersions (0.05 mg·mL^−1^ NPs) at room temperature and were recorded on a Bruker ELESYS spectrometer operating at the X-band. The spectrometer was equipped with a super-high-Q resonator, ER-4123-SHQ, the magnetic field was calibrated using a NMR probe and the frequency inside the cavity (~9.36 GHz) was determined with an integrated MW-frequency counter.

SAR (specific absorption rate) measurements were performed using a home-made AC magnetometer based on a previously described setup [81] that works in a frequency range (of 50–500 kHz) with variable field intensities: up to 80 mT at the low frequency limit and up to 45 mT at the high frequency limit. SAR values were obtained from the AC hysteresis loop area according to (1) expression. 

A resonant circuit fed by a radiofrequency (RF) power amplifier (Electronic & Innovation, mod. 1240 L) generated the external magnetic field, while the dynamic magnetisation was measured using an AC magnetometry pick-up coil system [78]. The AC magnetic field frequency was 311 kHz, whereas the field intensity was increased to 40 kA/m (400 Oe). The dynamic magnetisations were measured with the NP samples (≈1 mg mL^−1^) dispersed in water.

#### 3.2.5. Cytotoxicity Assay

In vitro cytotoxicity assays were performed on the HTC116 cell line (ATCC, American Type Culture Collection), using a (3-(4,5-dimethylthiazol-2-yl)-2,5-diphenyltetrazolium bromide) tetrazolium reduction (MTT) assay. Briefly, 7000 HTC116 cells were seeded per 96-well plate and incubated at 37 °C in a humidified atmosphere containing 5% CO_2_ for 24 h in supplemented Dulbecco’s modified Eagle medium (DMEM). Then, five different concentrations of A and B NPs functionalised with PD, PD-PEG, PD-EM102 and PD-EM102-PEG samples (0–525 µg·mL^−1^) were added to each well and the plates were again incubated for 24 h. Cells without nanoparticle treatment were used as a control. After incubation, 20 µL of MTT (0.5 mg·mL^−1^ in DMEM) was added to each well and the plates were incubated for 4 h. Then, 100 µL of DMSO was added after removing the medium from each well, and the plates were gently shaken for 30 min. Cell viability was determined using a microplate reader (iEMS Reader MS, Labsystems, Bradenton, FL, USA) at 570 nm. The relative cell viability (%) related to negative control wells containing cells without NPs was calculated by comparing the absorption of the medium of cells exposed to NPs, A_test_, versus the absorption of the medium of control cells, A_control_.

(2) $$\mathrm{Cell}\;\mathrm{viability}=\frac{A_{\mathrm{test}}}{A_{\mathrm{control}}}*100\%\;$$

## 4. Conclusions

In this study, we presented the preparation of multifunctional nanosystems based on Fe_3_O_4_ magnetic NPs functionalised with dodecylamine, a selenium-based drug and PEG-amine modified PMA copolymers. The thermal decomposition of iron(III) oleate precursor yielded NPs of a 13 ± 1 and 18 ± 1 nm average size with cuboctahedral and octahedral morphologies. Specifically, the relation of benzyl ether to 1-octadecene solvent volumes allows for the control of NPs sizes. Furthermore, the synthesis temperature can also affect particle size and shape. The amount of oleic acid in the synthesis solution influences nucleation and the growing processes and prevents the formation of specific morphologies of NPs. In this context, NPs with magnetic saturation values of 92 and 78 Am^2^ kg^−1^ were obtained. The high-saturation magnetisation value obtained for the Fe_3_O_4__B ferromagnetic sample is due to the stoichiometry of these NPs, as was corroborated by the highest Verwey transition temperature. The heating power of Fe_3_O_4__A@PD-PEG and Fe_3_O_4_ _B@PD-PEG was analysed and SAR values of 109 W·g^−1^ and 775 W·g^−1^ (the mass refers to the NP cores) at 305 kHz were recorded, the latter being potentially suited for applications in therapies based on magnetic hyperthermia. NPs functionalised with selenium-containing EM102 drug-modified PMA copolymers were also studied as potential drug carries, facilitating the incorporation of a high content of drug immobilised on the surface of the NPs. An optimisation of the PEG quantity for each nanoparticle sample was performed. The results obtained from in vitro tests carried out with the nanosystems conjugated with the drug proved their cytotoxicity in the HTC-116 cell line, being more representative in the case of nanosystems with the Fe_3_O_4__A@PD-EM102 NPs. 

## Figures and Tables

**Table 1:** Reagent amounts of iron(III) oleate, oleic acid, octadecene (ODE) and dibenzyl ether (DBE) used in the synthesis of the NPs, XRD peak position of the (311) maximum, particle crystalline core diameters calculated from the Scherrer formula (D_XRD_), particle core diameters observed from TEM (D_TEM_), and content of organic matter obtained via thermogravimetry (% OM).

Sample	Iron(III) Oleate (mmol)	Oleic Acid (mmol)	ODE:DBE (mL)	T_reflux_ (°C)	(311) Peak pos. (2θ)	D_XRD_ (nm)	D_TEM_ (nm)	% OM
**Fe_3_O_4__A**	5	10	10:10	304–295	35.661	11 ± 1	13 ± 1	12
**Fe_3_O_4__B**	5	10	12:06	312–297	35.637	18 ± 1	18 ± 2	10

**Table 2:** Summary of particle diameters observed via TEM (D_TEM_), saturation magnetisation (M_s_), reduced remanence (M_r_/M_s_) and coercivity (H_c_) obtained from the hysteresis loops at 300 K and 5 K, gyromagnetic effective values (g_eff_) and peak-to-peak linewidth values (ΔH_pp_) calculated from EMR measurements.

Sample	D_TEM_ (nm)	M_s_ RT (Am^2^kg^−1^)	M_s_ 5 K (Am^2^kg^−1^)	M_r_/M_s_ 5 K	H_c_ 300 K (mT)	H_c_ 5 K (mT)	$g_{eff}$	ΔH_pp_ (Gauss)
Fe_3_O_4__**A**	13 ± 1	78	113	0.31	0.26	18	3.66	304
Fe_3_O_4__**B**	18 ± 2	92	108	0.43	0.75	40	3.32	458

**Table 3:** Summary of ICP-AES results, hydrodynamic diameters in distilled water (D_water_) and PBS (1:10) water solution (D_PBS_) and zeta potential (ζ) in water obtained for Fe_3_O_4__A and Fe_3_O_4__B NPs functionalised with PD, PD-PEG, PD-EM102 and PD-EM102-PEG copolymers.

Sample	Fe Content ng/mL	Se Content ng/mL	Number of Drug Molecules/NP	D_water_ (nm)	D_PBS_ (nm)	ζ (mV)
Fe_3_O_4__A@PD	3987	<0.1	-	52 ± 19	36 ± 6	−49
Fe_3_O_4__A@PD–PEG12.5%	3505	<0.1	-	54 ± 6	58 ± 1	−41
Fe_3_O_4__A@PD–EM102	4971	33	349	34 ± 14	32 ± 7	−50
Fe_3_O_4__A@PD–EM102–PEG	4613	10.8	123	55 ± 8	49 ± 12	−45
Fe_3_O_4__B@PD	3676	<0.1	-	100 ± 29	71 ± 30	−50
Fe_3_O_4__B@PD–PEG25%	3835	<0.1	-	62 ± 17	46 ± 19	−43
Fe_3_O_4__B@PD–EM102	4162	6.8	63	48 ± 8	45 ± 2	−51
Fe_3_O_4__B@PD–EM102–PEG	5084	20.5	154	73 ± 15	45 ± 18	−47

## Data Availability

Not applicable.

## Supplementary Materials

The following supporting information can be downloaded at https://www.mdpi.com/article/10.3390/ph16070949/s1. Figure S1: Deconvolution of the main diffraction peak (311) of the A and B samples. Figure S2: EDS of the NPs. Figure S3: Thermogravimetric analysis of the A and B nanoparticle samples; Figures S4–S8: Infrared spectra of the A and B samples functionalised with oleic acid and prepared PD, PD-PEG, PD-EM102 and PD-EM102-PEG ligands; Figure S9: Distribution of the hydrodynamic diameter obtained using DLS of the A and B samples functionalised with PD, PD-PEG, PD-EM102 and PD-EM102-PEG ligands in a water and saline medium. Table S1: Amounts of reagents used in the preparation of the copolymers. Expression E1: Crystallite size from Fe_3_O_4_ nanoparticle calculation; Expression S2: g_eff_ determination.
