# Supplementary material for: Preparation of Selenium-Based Drug-Modified Polymeric Ligand-Functionalised Fe3O4 Nanoparticles as Multimodal Drug Carrier and Magnetic Hyperthermia Inductor
Source: Pharmaceuticals (Basel). 2023 Jun 30;16(7):949. doi: 10.3390/ph16070949 (PMC10385492; doi:10.3390/ph16070949)
Supplement: Supplementary file 1 [file pharmaceuticals-16-00949-s001.zip › pharmaceuticals-2437337-supplementary.pdf]

Table S1. Amounts of reagents used in the preparation of the copolymers.

Figure. S1. Deconvolution of the main diffraction peak (311) of the A and B samples.

Figure. S2. Electron Diffraction Pattern from selected area of sample Fe_3O_4_A (left) and Fe_3O_4_B (right).

Figure. S3. Thermogravimetric analysis of the A and B nanoparticle samples.

Figure. S4- Fig. S8 Infrared spectra of the A and B samples functionalized with oleic acid and prepared PD, PD-PEG, PD-EM102 and PD-EM102-PEG ligands.

Figure. S9 Distribution of the hydrodynamic diameter obtained by DLS of the A and B samples, functionalized with PD, PD-PEG, PD-EM102 and PD-EM102-PEG ligands in water and saline medium.

Expression 1. Crystallite size of Fe_3O_4 nanoparticles (Scherrer Equation)

Expression 2. g_{eff} determination

Table S1. Amounts of reagents used in the preparation of the copolymers.

Sample	PMA (mmol)	DDA (mmol)	Triethylamine (mmol)	Drug (mmol)	PEG (mmol)	Solvent	Solvent (mL)
**PD**	6.4	4.8	7.17	-	-	THF	20
**PD – EM102**	0.676	0.507	0.676	0.03	-	DMF	20
**PD – PEG 12.5%**	0.064	0.048	-	-	0.0081	$CHCl_3$	2
**PD – PEG25%**	0.064	0.048	-	-	0.0162	$CHCl_3$	2
**PD – EM102 – PEG12.5%**	0.022	0.016	-	-	0.0028	$CHCl_3$	2
**PD – EM102 – PEG25%**	0.025	0.018	-	-	0.0031	$CHCl_3$	2

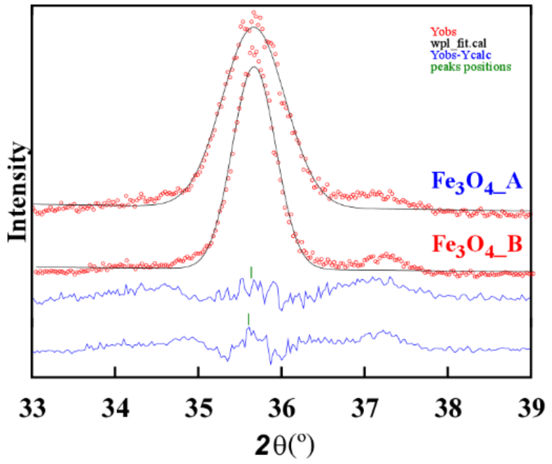

Figure. S1. Deconvolution of the main diffraction peak (311) of the Fe_3O_4_A and Fe_3O_4_B samples: experimental points (in red) = y_{obs}, peak position (turquoise mark) = peak pos, and difference between experimental data and fit (gray line) = $y_{obs} - y_{calc}$.

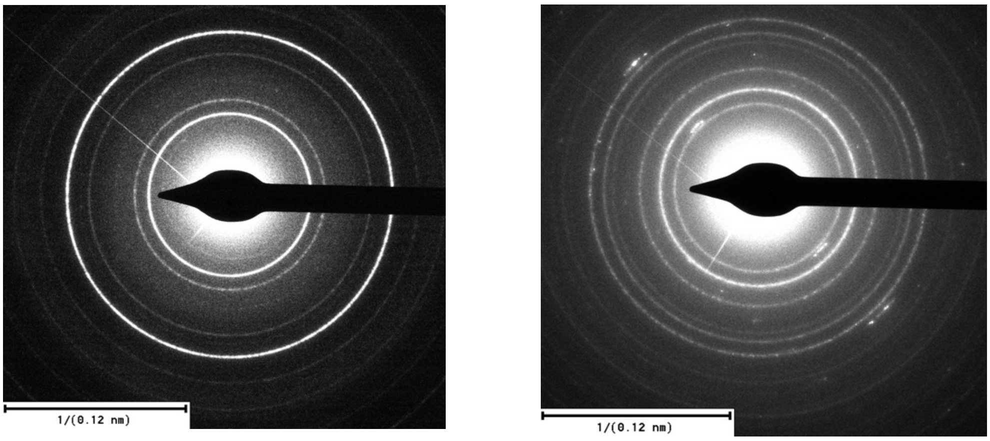

Figure. S2. Electron Diffraction Pattern from selected area of sample Fe_3O_4_A (left) and Fe_3O_4_B (right).

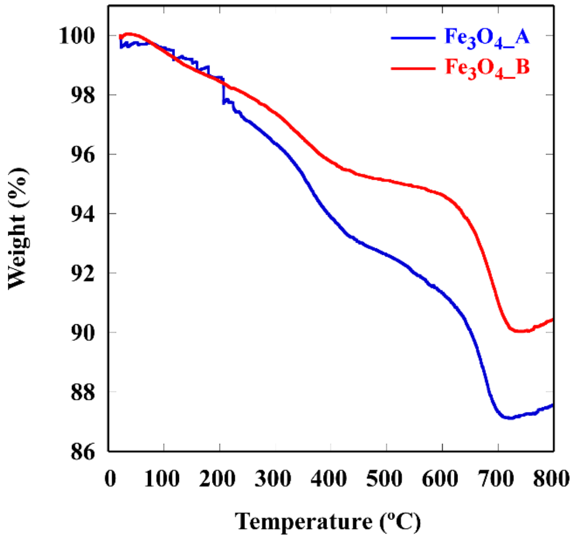

Figure. S3. Thermogravimetric analysis of the Fe_3O_4_A and Fe_3O_4_B nanoparticle samples.

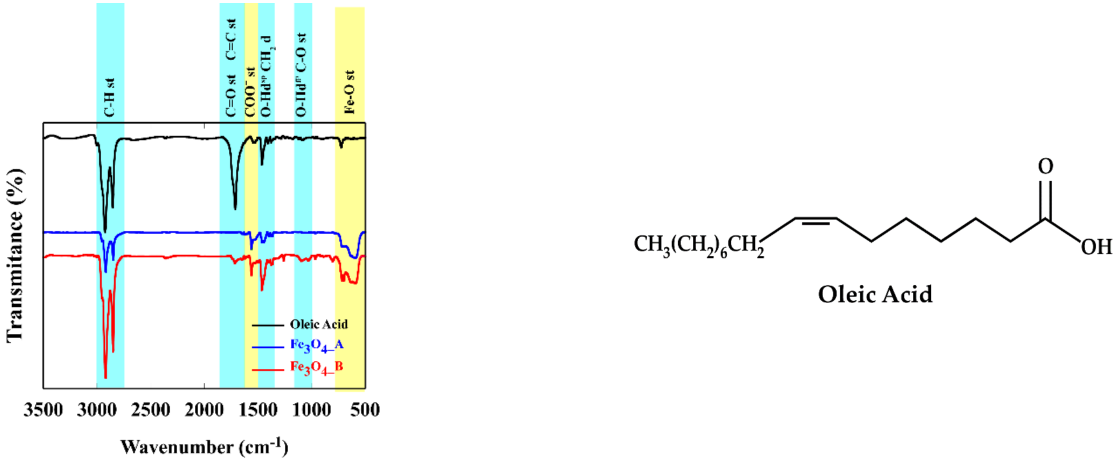

Figure. S4 Infrared spectra of oleic acid and the Fe_3O_4_A and Fe_3O_4_B samples in the left. The molecular structure of oleic acid is shown on the right side.

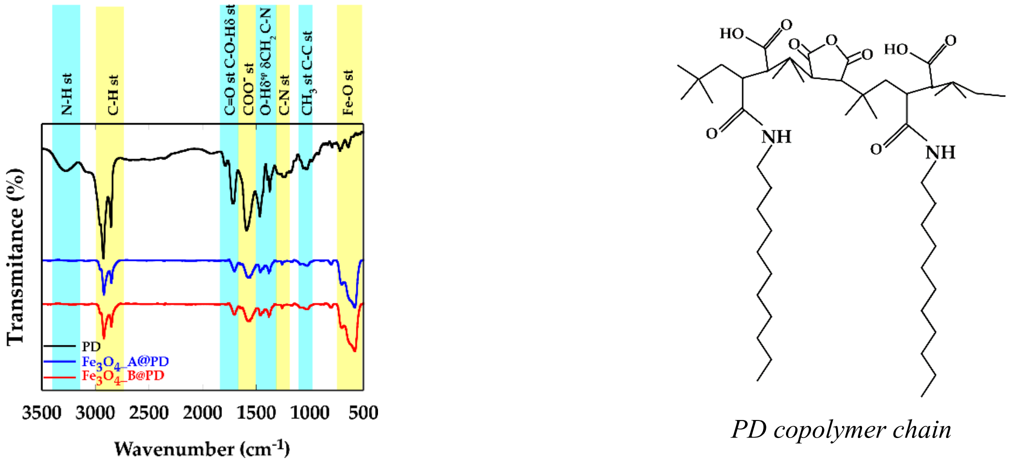

Figure. S5 Infrared spectra of PD copolymer and the Fe_3O_4_A-PD and Fe_3O_4_B-PD samples on the left. The molecular structure of the PD copolymer is shown on the right side.

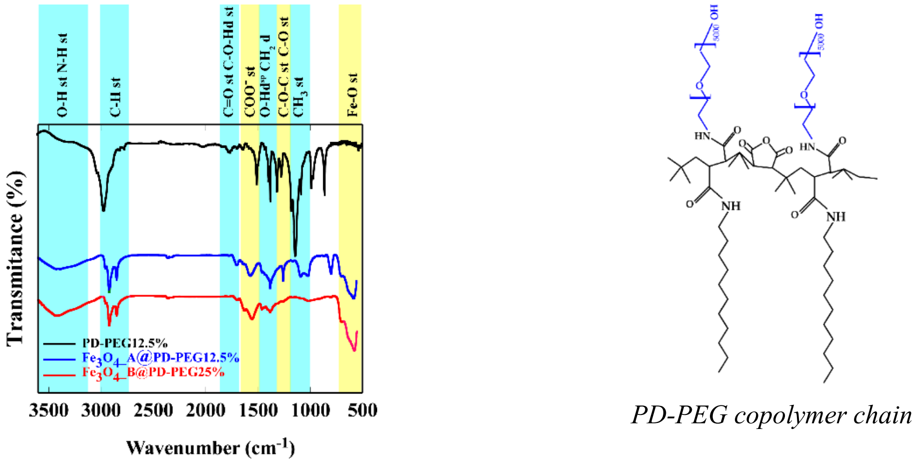

Figure. S6 Infrared spectra of PD-PEG12.5% copolymer and the Fe_3O_4_A-PD-12.5% and Fe_3O_4_B-PD-PEG25% samples in the left. The molecular structure of the PD-PEG% copolymer is provided on the right side.

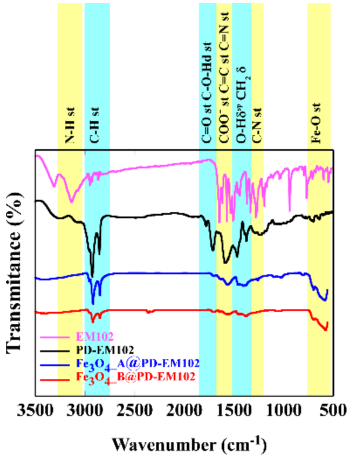

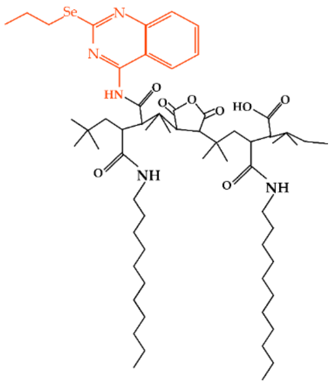

PD-EM102 copolymer chain

Figure. S7 Infrared spectra of drug EM102, PD-EM102 co-polymer, and the Fe_3O_4_A@PD-EM102 and Fe_3O_4_B@PD-EM102 samples on the left. The molecular structure of the PD-EM102 copolymer is shown on the right side.

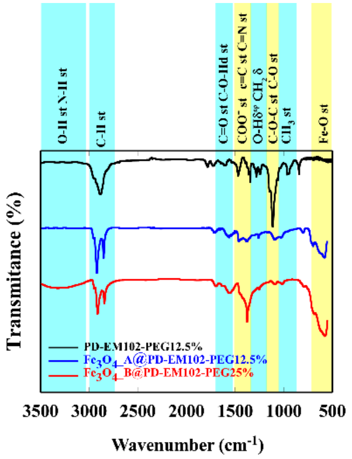

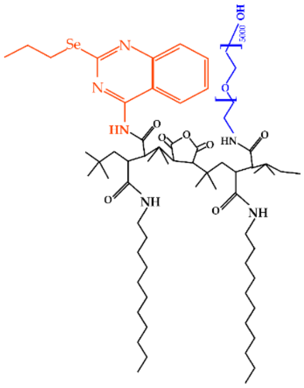

PD-EM102-PEG copolymer chain

Figure. S8 Infrared spectra of PD-EM102-PEG12.5% copolymer, and the Fe_3O_4_A@PD-EM102-PEG12.5% and Fe_3O_4_B@PD-EM102-PEG25% samples on the left. The molecular structure of the PD-EM102-PEG co-polymer chain fraction is shown on the right side.

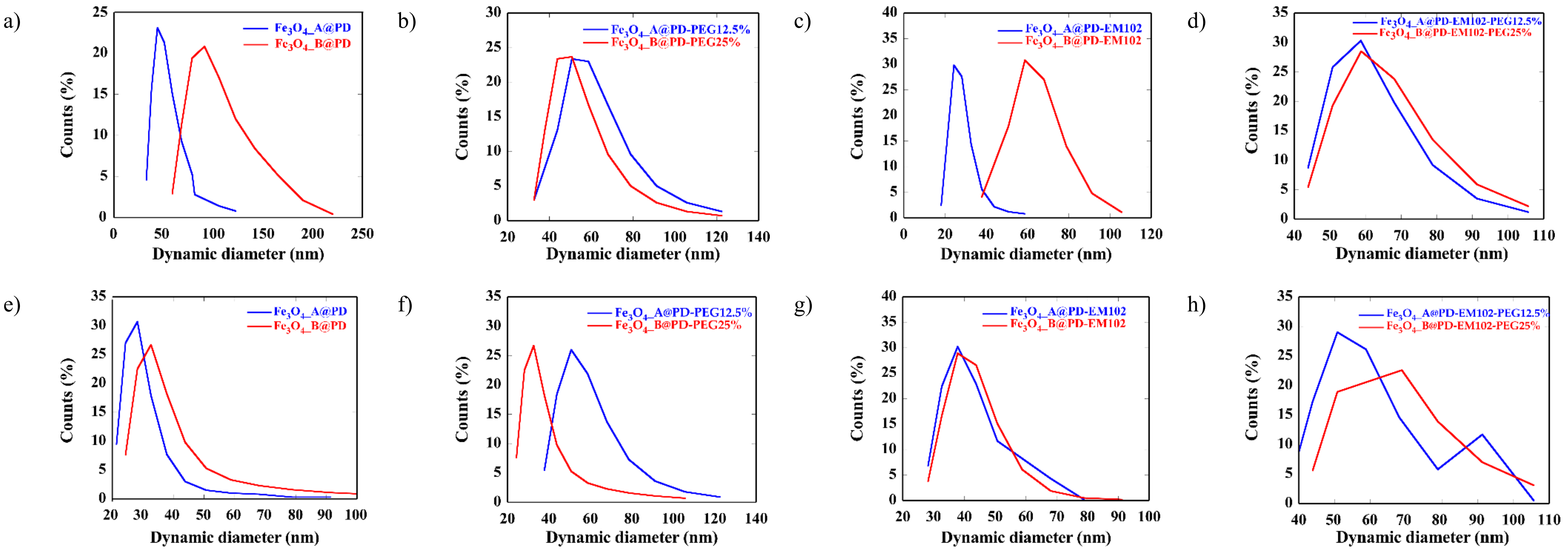

Figure. S9 Distribution of the hydrodynamic diameter obtained by DLS for the Fe_3O_4_A and Fe_3O_4_B nanoparticle samples functionalized with PD, PD-PEG, PD-EM102 and PD-EM102-PEG copolymers in water (a-d) and PBS (c-h) dispersion.

E1. Crystallite size of Fe_3O_4 nanoparticles

The crystallite sizes of samples A and B nanoparticles have been calculated by the deconvolution of the (311) diffraction peak of magnetite, using the Scherrer equation (S1):

$$D = \frac{K\lambda}{B_{structure}\cos\theta} \quad \text{(S1)}$$

Where K is the shape factor (0.85-0.95), $B_{structure} = B_{observed} - B_{instrumental}$ is the full width at half maximum, λ is the X-ray wavelength (in our case = $(K\alpha_1+K\alpha_2)/2=1.5418$ Å), and θ is the angle of the peak position.

E2. g_{eff} determination

In order to compare the observed EMR signals, a value of g_{eff} has been determined (S2) for each sample assuming that the resonant field corresponds to the maximum of the microwave absorption curve.

$$\Delta E = h\nu = g\mu_B H_r \rightarrow g_{eff} = \frac{h\nu}{\mu_B H_r} \quad \text{(S2)}$$

where h is the Plank's constant, ν is the microwave frequency, μ_B is the Bohr magneton ($9.27 \cdot 10^{-21}$ erg G^{-1}) and H_r is the resonant field.
